# Isolation, Characterization, and Evaluation of a Lytic Jumbo Phage Z90 Against *Aeromonas hydrophila* in American Eels (*Anguilla rostrata*)

**DOI:** 10.3390/antibiotics15010027

**Published:** 2025-12-31

**Authors:** Miaosen Zhang, Xuejin Feng, Jianxin Wang, Wu Qu, Min Jin

**Affiliations:** 1 Marine Science and Technology School, Zhejiang Ocean University, Zhoushan 316022, China; zhangmiaosen@zjou.edu.cn (M.Z.);; 2 State Key Laboratory Breeding Base of Marine Genetic Resource, Third Institute of Oceanography, Ministry of Natural Resources, Xiamen 361000, China; 3 Fujian Ocean Innovation Center, Xiamen 361102, China

**Keywords:** *Aeromonas hydrophila*, jumbo phage, biological characteristics, phage therapy, American eels

## Abstract

**Background:** *Aeromonas hydrophila* is a common bacterial pathogen that causes hemorrhagic septicaemia in several farmed aquaculture species. Phage therapy is considered a promising and feasible alternative to antibiotic treatment. **Methods:** In this study, an *A. hydrophila*-infecting jumbo phage Z90 was isolated from an aquaculture pond. The biological characteristics, genomic features, and in vitro and in vivo experiments were investigated to evaluate its application potential. **Results:** Phage Z90 was a myovirus with distinctive curled tail fibers. Additionally, phylogenetic and genomic analyses found that the phage Z90 was a novel virus belonging to the genus *Ferozepurvirus* of the family *Chimalliviridae*. One-step growth curve analysis revealed that the phage Z90 was a lytic phage, exhibiting a short latency period of 20 min and a relatively large burst size of 270 ± 42 PFU/cell. The phage Z90 particles were stable at psychrotrophic and mesophilic temperatures (10–50 °C) and a wide range of pH (pH 3–12). Genomic analysis revealed that the phage Z90 did not contain any genes encoding toxins, virulence factors, or antibiotic resistance factors. In vivo analysis demonstrated that the phage Z90 protected American eels from *A. hydrophila* infection, greatly increasing eel survival rates and alleviating symptoms caused by bacterial infections. The comparison of different phage administration methods suggested that phage Z90 was better administered through intraperitoneal injection than immersion in aquaculture water. Moreover, the combination of phage Z90 and ampicillin improved the bactericidal effect and reduced the treatment dosage compared to antibiotics or phage alone. **Conclusions:** Altogether, the findings of this study indicate that the phage Z90 can serve as a promising biocontrol agent for the treatment of *A. hydrophila* infection in aquaculture.

## 1. Introduction

Eel (*Anguilla* spp.) is commonly known as “underwater ginseng” [1] for its delicious meat, rich nutrition, and medicinal value [2]. It is a widely cultured fish species globally, with over 45 countries engaged in eel production. In recent decades, eel production has seen remarkable outputs, reaching a total production of 277,103 tonnes and 2.67 billion eels in trade internationally [3]. As a high-value species, eels command premium prices in global markets and play a significant role in the fisheries economy. However, intensive high-density aquaculture practices, essential for meeting commercial demand, render cultivated eels highly susceptible to bacterial infections, which can lead to mass mortality events and severe economic losses. Notably, studies have reported that bacterial diseases may account for up to 90% of production losses in eel aquaculture, making them a major contributor to the sector’s economic burden [4,5].

*Aeromonas hydrophila* (*A. hydrophila*) is a Gram-negative bacterium widely distributed in aquatic environments, sediments, and various food sources [6]. It is a major bacterial pathogen in global aquaculture and causes hemorrhagic septicemia in multiple commercially important fish species, including carps, tilapia, perch, catfishes, salmon, and eels [7,8], resulting in immense economic losses to the freshwater aquaculture industry worldwide [9,10]. Notably, in intensive eel farming systems, *A. hydrophila* is recognized as a primary etiological agent of highly contagious diseases, such as hemorrhagic septicemia and gill rot, particularly in European eels (*Anguilla anguilla*), Japanese eels (*A. japonica*), and American eels (*A. rostrata*), with peak incidence observed during the spring–summer transition period [11].

Antibiotics are routinely employed to prevent and treat bacterial infections in aquaculture systems. However, the excessive use of antibiotics has led to the dissemination of antibiotic resistance genes and the emergence of multidrug-resistant (MDR) bacterial strains [12], jeopardizing the sustainability of aquaculture systems and posing significant risks to food safety and public health. In recent years, bacteriophages have emerged as a powerful alternative to combat MDR strains, owing to their host specificity and versatile applicability [13]. Jumbo phages, defined as DNA phages with genomes larger than 200 kb [14], possess rich genetic content that enables the expression of multiple functionally important proteins, including endolysins, virion-associated lytic enzymes, and polysaccharide depolymerases [15,16]. These lysis-associated enzymes efficiently degrade bacterial cell walls and biofilm matrices, enhancing the phage’s lytic performance and making jumbo phages promising candidates for combating bacterial resistance [17,18,19]. Beyond their extensive enzymatic arsenal, jumbo phages have evolved innovative anti-defense strategies, including self-assembled phage nucleus structures that compartmentalize replication from bacterial immunity systems [20,21]. These evolutionary adaptations position jumbo phages as next-generation antimicrobial reagents capable of overcoming pan-drug-resistant pathogens. Furthermore, jumbo phages possess unique genomic features that confer greater independence from host molecular machinery compared to other phages, which likely underlies their broad host ranges [22]. Specifically, their genomes encode phage-encoded RNA polymerases (RNAPs) [23,24] for early gene transcription, and non-virion RNAPs for middle and late gene transcription [25], allowing transcriptional processes to proceed independently of the host. In addition, jumbo phages harbor a repertoire of tRNAs and aminoacyl-tRNA synthetases that can substitute for cleaved host tRNAs. Their genomes also contain numerous genes involved in DNA replication, modification, and nucleotide metabolism, often organized into sub-clusters [22,26,27]. Collectively, these features enable autonomous replication and viral protein production,, reducing reliance on host systems and enhancing their adaptability and infection efficiency across diverse hosts.

Numerous studies have demonstrated that phages can reduce or eliminate the mortality rates of aquaculture animals caused by bacteria [28,29,30,31,32]. However, reports on the use of jumbo phages in aquaculture remain limited [33]. Moreover, phage therapy studies have not yet been reported for American eel, which is the primary eel species farmed in China [11]. In this study, we isolated a lethal *A. hydrophila* pathogen from diseased American eels and further isolated its lytic jumbo phage Z90 from aquaculture water. Comprehensive one-step growth curve, genomic, and environmental stability analyses were conducted to evaluate the biological characteristics of the phage Z90. Furthermore, we extensively evaluated the in vitro and in vivo therapeutic potential of the phage Z90 against *A. hydrophila* infection in American eels. Moreover, we also evaluated different administration methods (intraperitoneal injection and immersion) for phage therapy in American eel, establishing a critical foundation for practical phage therapy in eel aquaculture systems.

## 2. Results

### 2.1. Isolation and Identification of A. hydrophila 2408

Nine bacterial strains (Supplementary Table S1) were isolated from the rotten skin and abdominal hemorrhagic fluid of diseased American eels (*Anguilla rostrata*). 16S rRNA gene sequencing showed that one of the isolated bacterial strains shared 99% similarity with *A. hydrophila,* a known lethal pathogen to eels [11]. This isolate formed yellow colonies with neat margins on Rimler–Shotts (RS) medium (Supplementary Figure S1). Phylogenetic analysis based on 16S rRNA sequences (Supplementary Figure S2) and whole-genome sequences (Supplementary Figure S3) clustered this isolate with known *A. hydrophila* strains. Genome-wide comparisons further revealed high digital DNA–DNA hybridization values with *A. hydrophila* ATCC 7966 (dDDH d0 = 91.7%, dDDH d6 = 91.5%) and a minimal G + C content difference (0.22%), supporting their close relatedness. Collectively, these results classify the isolate as *A. hydrophila*, and it was designated as *A. hydrophila* 2408. A total of 691 virulence-associated genes were identified in the genome of *A. hydrophila* 2408 (Table 1), including those encoding aerolysin (*aerA*), hemolysin (*hlyA*), exotoxin A (toxA), flagella (*fla*), and thermostable hemolysin (*tlh*) [8]. The high abundance and complexity of virulence factor genes in *A. hydrophila* 2408 genome provide genomic evidence for its high pathogenicity towards eels and suggest potential multifactorial pathogenic mechanisms.

### 2.2. Morphological Characteristics of the Phage Z90

**Figure 1:**
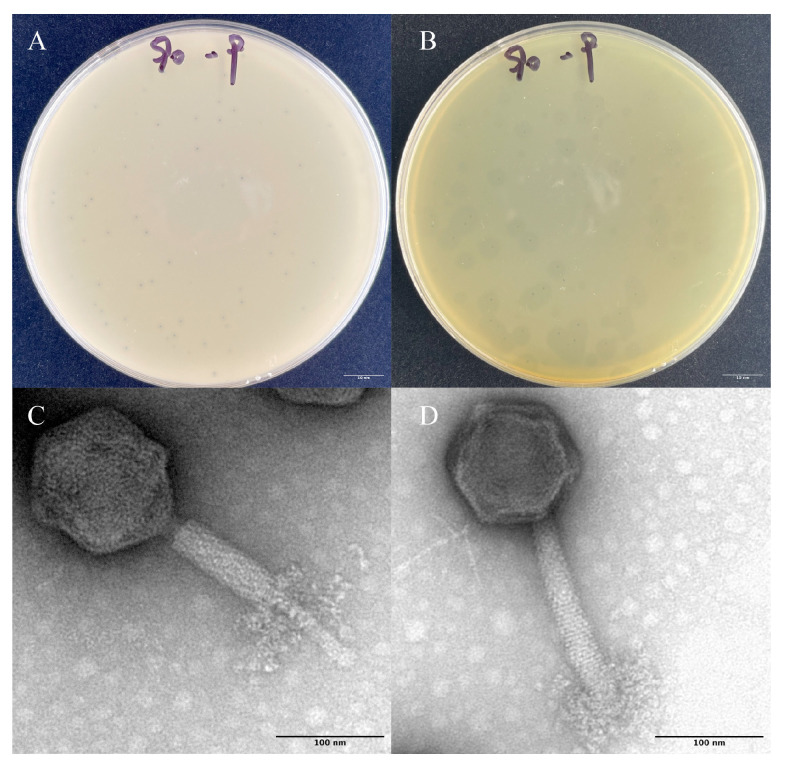
Morphological characteristics of the phage particles and plaques: (**A**) Plaque morphology after 12 h of incubation. (**B**) The halo zone surrounding the plaques after 24 h of incubation. (**C**,**D**) Phage particles consisting of a contractile tail and distinctive curled-tail fibers. Scale bar, 100 nm.

The phage Z90 was isolated from the aquaculture pond in Xiamen, using *A. hydrophila* 2408 as the host. The phage Z90 produced clear dot-like small plaques of 1–1.6 mm diameter on double-layer agar plates (Figure 1A). After further incubations, the phage produced a halo zone of 8.5 mm diameter around plaque. The halo zone surrounding plaque was believed to be related to the ability of phage components to depolymerize exo-polysaccharides [34]. TEM analysis showed that the phage Z90 was a typical myovirus, consisting of a polyhedral head of 53 ± 2 nm diameter and a contractile tail of approximately 85 ± 5 nm, with distinctive curled-tail fibers.

### 2.3. Host Range of the Phage Z90

A total of 15 bacterial strains were used to evaluate the host range of the phage Z90 (Table 2). The results demonstrated that the phage Z90 exhibited specific lytic activity against *A. hydrophila* 2408 among the tested bacteria, indicating a relatively narrow host range. This host specificity of phage Z90 may be attributed to either the limited number of *Aeromonas* strains tested or the unique specificity of the host recognition site for phage Z90.

### 2.4. One-Step Growth Curve Analysis of the Phage Z90

**Figure 2:**
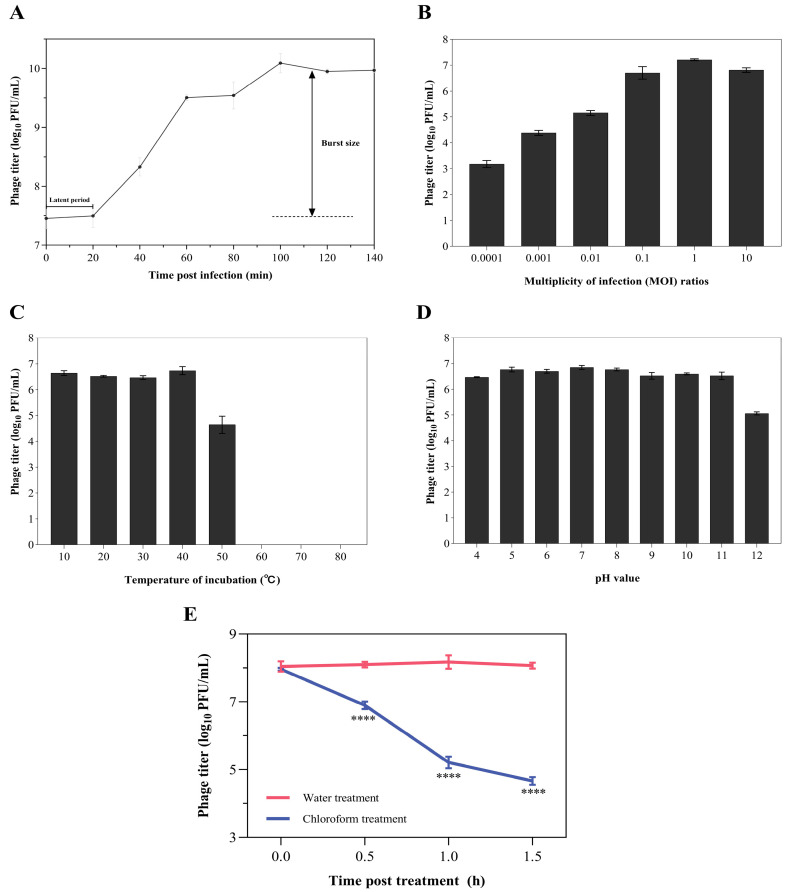
Biological characteristics of the phage Z90 (**A**) One-step growth curve analysis of the phage Z90. (**B**) Optimal MOI of the phage Z90. (**C**–**E**) Temperature (**C**), pH (**D**), and chloroform (**E**) stability of the phage Z90. Values are expressed as the mean ± standard deviation from three biological replicates. For chloroform stability (**E**), phage titers at each time point were compared between water and chloroform treatment groups using one-way ANOVA; **** *p* < 0.0001 indicates significant reduction compared with the water treatment.

The life cycle of phage Z90 was determined using the one-step growth curve analysis (Figure 2A). The results showed that the phage Z90 exhibited a latent period of approximately 20 min post-infection, followed by a rapid increase in phage titer that reached a plateau at approximately 100 min. Additionally, the phage Z90 demonstrated a relatively large burst size of approximately 270 ± 42 PFU per infected cell.

### 2.5. Optimal MOI and Temperature, pH, and Chloroform Stability of the Phage Z90

Different MOIs (10^−3^, 10^−2^, 10^−1^, 1, and 10) were examined to determine the optimal MOI for phage infection and replication. The results showed that the phage infection efficiency was the highest at an MOI of 1 (Figure 2B). Therefore, all subsequent stability assays were performed at this MOI. The incubation of phage particles at 10–40 °C for 1 h did not greatly affect the phage titer (~10^6^ PFU/mL, Figure 2C) However, incubation at 50 °C reduced the phage titer to 10^4^ PFU/mL, while incubation at 60–80 °C terminated phage activity (Figure 2C). The phage particles showed excellent stability at pH values ranging between 4 to 11 (phage titer ~10^6^ PFU/mL); however, their titer dropped to 2 × 10^5^ PFU/mL at pH 12 (Figure 2D). Chloroform treatment (5% *v*/*v*) significantly reduced phage titer over time (Figure 2E), suggesting that phage particles may be composed of a lipid-based exterior.

### 2.6. Genomic Characteristics of the Phage Z90

**Figure 3:**
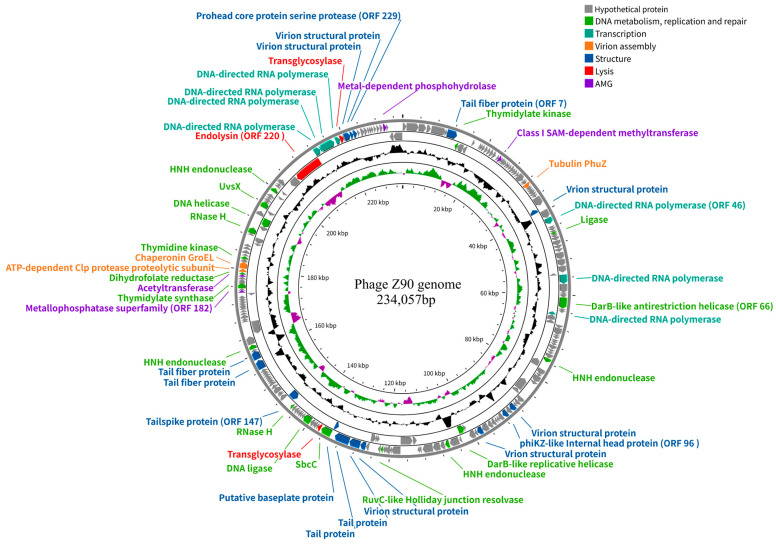
Genome map of the phage Z90. From outer to inner circles: (1) the functionally annotated ORFs (arrows indicate the direction of transcription and colors represent different functional modules); (2) G  +  C% content; (3) GC skew plot; and (4) genome position.

The phage Z90 genome consists of dsDNA of 234,057 bp, with 38.4% G + C content, indicating that it is a jumbo phage (>200 kb, Figure 3). It contains three putative tRNA-encoding genes and 242 ORFs, of which 50 were annotated with putative functions based on their amino acid sequence and conserved domains. The predicted functional ORFs were classified into different categories, including DNA metabolism and replication, transcription, virion assembly, structural proteins, lysis, and auxiliary metabolic genes (AMGs). Notably, the phage genome did not contain any known genes associated with virulence factors or antibiotic resistance.

The Z90 genome encoded a large number of ORFs related to DNA metabolism, replication, and repair, including thymidylate kinases (ORFs 8, 184, and 195), DNA ligase (ORF 139), DNA helicases (ORFs 107 and 205), HNH endonucleases (ORFs 83, 110, 165, and 214), and RNase H enzymes (ORFs 146 and 203). In addition, the Z90 genome encoded abundant ORFs associated with transcription, including DNA-dependent RNA polymerases (RNAPs; ORFs 46, 61, 69, and 221–224). The high abundance of DNA-dependent RNA polymerase imply that the phage Z90 may rely primarily on its own RNAP for transcription, which is typical for phiKZ-like jumbo phages [35]. Furthermore, the Z90 genome encoded several other predicted ORFs, such as ORF066 (encoding a DnaB-like replicative helicase), ORF133 (encoding a SbcCD complex ATPase involved in DNA repair), and ORF146 (encoding a RNase H involved in DNA repair), which are considered as core components of the jumbo phage genomes [14].

Approximately 30% of the functionally annotated ORFs were predicted to be involved in structural and assembly processes, including those encoding structural proteins, like the tail fiber protein (ORF 007), tubulin PhuZ (ORF 035), phiKZ-like internal head protein (ORF 096), tail spike proteins (ORFs 147, 162, and 163), tail proteins (ORFs 130 and 131), and baseplate protein (ORF 132). The phiKZ-like internal head structure, known as the inner body, is considered an essential feature of the phiKZ-like jumbo phages. Although its functions have not been experimentally verified, the inner body is hypothesized to be multifunctional, potentially contributing to DNA packaging, genome organization, DNA ejection, and phage development. Recent evidence suggests that proteins associated with the inner body may participate in dynamic structural rearrangements during phage head maturation and genome encapsidation. The PhuZ, ATP-dependent Clp protease (ORF 191), and chaperonin GroEL (ORF 192) are classified as virion assembly-related ORFs, with PhuZ functioning as a tubulin-like protein that forms dynamic filaments to position the phage nucleus at the center of the bacterial cell and transport capsids for phage DNA packaging during infection [36].

Furthermore, a few functionally annotated ORFs were predicted to be involved in host lysis, including those encoding transglycosylases (ORF 134 and 225) and a putative endolysin (ORF 220). In addition, some ORFs were predicted to be involved in host metabolic reprogramming, including those encoding a Class I S-adenosylmethionine-dependent methyltransferase (ORF 020), metallophosphatase superfamily protein (ORF 182), and metal-dependent phosphohydrolase (ORF 239) [37].

### 2.7. Phylogenetic and Taxonomic Classification of the Phage Z90

**Figure 4:**
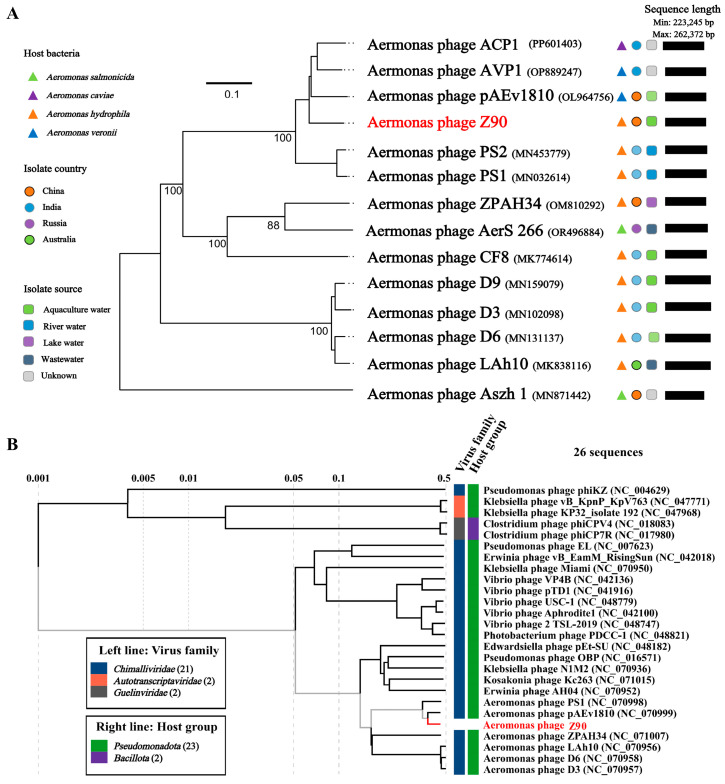
Phylogenetic analysis of the phage Z90 and its related phages (**A**) Phylogenomic tree = based on the Genome-BLAST distance phylogeny (GBDP) method generated using the Virus Classification and Tree Building Online Resource (VICTOR) tool with the D6 formula. (**B**) Proteomic tree of the phage Z90 and its related phages generated by Viptree. The viruses were classified according to the ICTV classification system (March 2025 release).

The genome-wide phylogenetic tree of the phage Z90 and its related phages, six phage genomes with top genome homology to phage Z90 were retrieved based on NCBI BLASTN analysis, including Aeromonas phage AVP1 (OP889247), Aeromonas phage pAEv1810 (NC_070999), Aeromonas phage ACP1 (PP601403), Aeromonas phage PS2 (MN453779), Aeromonas phage PS1 (NC_070998.1), and Aeromonas phage CF8 (MK774614). In addition, he genomes of *Aeromonas*-infecting jumbo phages were also included for the phylogenetic analysis. The results revealed that the phage Z90 clustered closely with pAEv1810, indicating a close evolutionary relationship between the two (Figure 4A). This clustering was further supported by the phylogenetic proteomic tree generated via the ViPTree server (Figure 4B).

**Figure 5:**
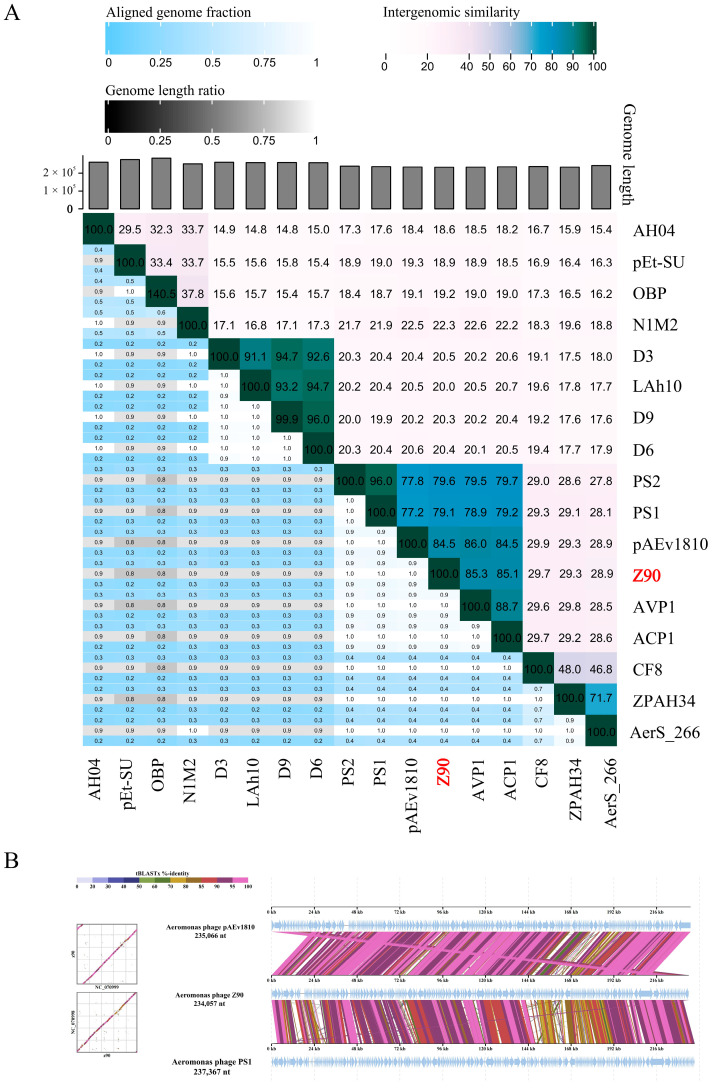
Intergenomic comparison of the phage Z90 and its related phages: (**A**) Intergenomic similarity between the phage Z90 and its related phages, calculated using VIRIDIC. (**B**) Genome comparison of the phage Z90, pAEv1810, and PS1.

Intergenomic similarity analysis between the phage Z90 and its related phages showed that the phage Z90 shared the highest average nucleotide identity (84.5%) with pAEv1810 (Figure 5A). Although the phages Z90 and pAEv1810 were isolated from aquaculture water, they were isolated from different bacterial strains (*A. hydrophila* and *A. veronii*, respectively). Furthermore, the phage Z90 was isolated from China, whereas pAEv1810 was isolated from Australia, suggesting a global distribution of these evolutionarily close phage species. Altogether, the phylogenetic and intergenomic similarity analyses suggest that the phage Z90 was a novel virus belonging to the genus *Ferozepurvirus* in the family *Chimalliviridae*.

### 2.8. In Vitro Bactericidal Effects of the Phage Z90

**Figure 6:**
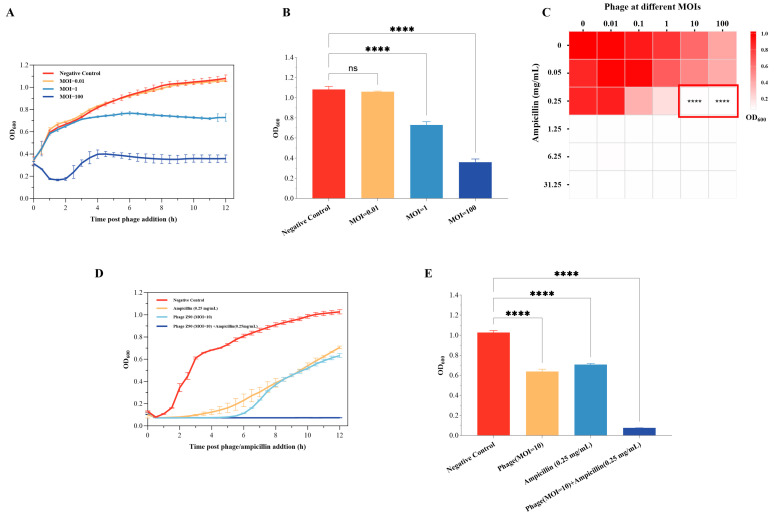
In vitro bactericidal effects of the phage Z90 against *A. hydrophila* 2408: (**A**) Killing curves showing the bactericidal effects of the phage Z90 against *A. hydrophila* 2408 at different MOIs. (**B**) The final OD_600_ values at 12 h post phage addition for different groups from panel (**A**). (**C**) Checkerboard screening assay showing the synergistic activities of the phage Z90 and ampicillin. Heatmap shows the OD_600_ values of the bacterial culture upon treatment with different combinations of the phage Z90 and ampicillin doses after 12 h of treatment. The red-outlined region indicates the combination treatments that achieved near-complete bacterial lysis (OD_600_ < 0.1). Statistical analysis revealed that these combination treatments significantly inhibited *A. hydrophila* 2408 compared with the blank control and each single treatment (**** *p* < 0.0001). (**D**) Killing curve showing the synergistic activity of the phage Z90 and ampicillin. (**E**) Final OD_600_ values at 12 h post phage/ampicillin addition for different groups from panel (**D**). Group differences were statistically assessed using one-way ANOVA followed by Tukey’s post-hoc multiple comparison test. ns = not significant; **** *p* < 0.0001.

The in vitro bactericidal effects of the phage Z90 against *A. hydrophila* 2408 at different MOIs were investigated using killing curve analysis (Figure 6A). The results showed that the phage Z90 exhibited in vitro bactericidal effects in a dose-dependent manner. At the MOI of 100, the phage Z90 caused strong lysis of the 2408 strain, effectively maintaining its OD_600_ below 0.4 throughout 12 h of incubation. At the MOI of 1, the phage Z90 demonstrated measurable bactericidal effects, reducing the OD_600_ by 31% at the end of incubation in comparison with the NC. In contrast, at the MOI of 0.01, the phage Z90 infection had little effect on bacterial growth.

Moreover, the in vitro bactericidal effect of the phage Z90 was explored in combination with ampicillin (Figure 6C). The results demonstrated that the combination of phage Z90 and ampicillin exhibited synergistic bactericidal effects. For instance, neither ampicillin (0.25 mg/mL) alone nor phage treatment (MOI = 10) alone was sufficient to effectively inhibit the growth of *A. hydrophila* 2408 (Figure 6C,D). However, their combined application at the same doses resulted in a near-complete lysis of bacterial cells and sustained suppression of bacterial regrowth throughout the 12-h incubation period (Figure 6D). These findings suggest that the phage Z90 can serve as a promising agent to control *A. hydrophila* 2408 in vitro,, especially in combination with antibiotics.

### 2.9. In Vivo Therapeutic Effects of the Phage Z90

**Figure 7:**
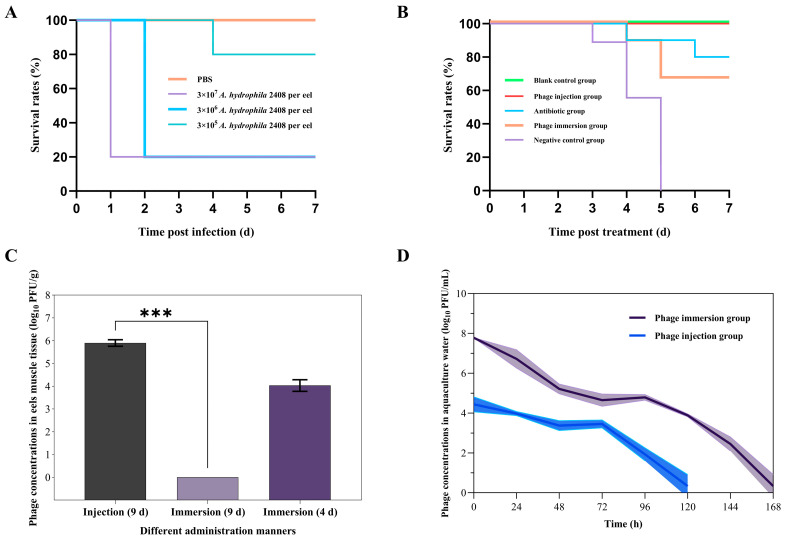
In vivo therapeutic efficacy of the phage Z90 against *A. hydrophila* 2408 infection in diseased eel model: (**A**) The establishment of *A. hydrophila*-infecting diseased eel model. Survival rate of eels after infection with *A. hydrophila* 2408 was shown. The PBS group received the injection of PBS, while other groups were injected with *A. hydrophila* 2408 at different concentrations. (**B**) Survival rates of diseased American eels after phage (immersion and intraperitoneal injection) and antibiotic treatments. In blank control group, healthy eels were not given any treatment. The eels in other four groups were first treated by intraperitoneal injection of 0.1 mL of *A. hydrophila* 2408 at a concentration of 6 × 10^6^ CFU/mL. Two hours after bacterial infection, eels in the negative control group, antibiotic group, and phage injection group were injected with 0.1 mL of PBS, 0.1 mL of ampicillin (2.5 mg/mL), and 0.1 mL phage Z90 (6 × 10^8^ PFU/mL), respectively. For the phage immersion group, phage concentrate was added to the aquaculture water to a final concentration of 10^8^ PFU/mL. The survival curves were plotted using the KaplanMeier method, and the log-rank test was used to analyze the difference in survival rates. (**C**) The phage Z90 concentrations in the muscle tissue of American eels in the phage injection and immersion groups. Statistical analysis was performed using the Mann–Whitney U test (***, *p* < 0.001). (**D**) The phage Z90 concentrations in the aquaculture water of the phage injection and immersion groups.

To establish the *A. hydrophila*-infected eel model, American eels were intraperitoneally injected with varying doses of *A. hydrophila* 2408. At 48 h post-injection, the 10^5^, 10^6^, and 10^7^ CFU/eel *A. hydrophila* 2408 injections led to 0%, 80%, and 80% mortality rate of the American eels, respectively (Figure 7A). In contrast, no deaths occurred in the PBS-injected control group within 7 d (Figure 7A). In addition, the American eels showed an increase in infection symptoms with an increase in injection dose, with the main symptoms being congested head, abdominal edema, swollen gallbladder filled with bile, bleeding in the pelvic fins, and reduced movements. After death, a high abundance of *A. hydrophila* 2408 was detected in the muscle tissue and internal tissues of the eels. These results indicate the successful establishment of *A. hydrophila*-infected eel model.

To further explore the in vivo therapeutic effects of the phage Z90, the *A. hydrophila* 2408-infected eel models were treated with the phage Z90, administered via intraperitoneal injection or immersion (Figure 7B). After treatment, the infected eels in the group exhibited marked behavioral lethargy, weak avoidance responses upon stimulation, and high mortality at 3 dpi and 100% mortality at 5 dpi (Figure 7B). In contrast, the phage immersion group achieved a 7d survival rate of >70%, comparable to that of the antibiotic group (80%). Notably, the phage injection group showed no mortality during the 7d observation period, similar to the blank control group (Figure 7B). To further examine the influence of the administration method on the accessibility and in vivo therapeutic efficacy of the phage Z90, the surviving eels from the phage injection and immersion groups were euthanized on day 9 using tricaine methanesulfonate (MS-222). The muscle tissues from the euthanized and dead eels were collected for phage quantification. The results showed that phage concentration in the muscles of the phage injection group remained high on day 9 (10^6^PFU/g), while that in the muscles of the phage immersion group was only 10^4^ PFU/g on day 4 and below the detection limit on day 9 (Figure 7C). Phage Z90 concentration in the muscles of the phage injection group was significantly higher than that in the muscles of the phage immersion group on day 9 (Mann–Whitney U test, *p* = 0.00016). In addition, phage concentrations in the aquaculture water of both the groups reduced over the 7d period (Figure 7D); However, the phage concentration in the phage immersion group was initially higher than that in the phage injection group but ultimately dropped below the detection limit. These findings indicate that intraperitoneal injection of the phage Z90 provides more sustained in vivo persistence and enhanced therapeutic efficacy compared to immersion, underscoring its superior therapeutic potential in aquaculture applications.

## 3. Discussion

*A. hydrophila* is a major pathogen in eel aquaculture and has been reported to infect Japanese eels [38] and European eels [39]. A recent study demonstrated its pathogenicity and host immune response in American eels [11]. As the major eel species cultured in China, American eels have a high economic value. Therefore, it is essential to isolate and characterize its pathogens to prevent potential diseases and subsequent economic losses. In this study, a highly pathogenic strain of *A. hydrophila* was isolated from diseased eels, and its virulence and pathogenicity towards American eels were further examined through its intraperitoneal injection in eels, which provided valuable genomic and physiological data to control *A. hydrophila* in aquatic animals.

Biological characterization and genetic safety evaluation of bacteriophages are essential for their practical application in aquaculture [40]. The burst size and latent period are critical factors influencing the therapeutic efficacy of phages [41], as a high burst size increases the likelihood of phages reaching target bacteria and accelerating host elimination, while a short latent period allows progeny phages to infect new host cells sooner and proliferate more rapidly [42]. Phage Z90 has a large burst size (270 ± 42 PFU/cell) compared to similar phages, while PZL-Ah152 produces only ~91 phage particles per cell and ZPAH34 yields 79 phages per cell [30,43]. The one-step growth curve analysis revealed that the phage Z90 was a lytic phage with a short latency period of 20 min, comparable to phages PZL-Ah152 and ZPAH34, yet markedly shorter than that of phage D6 (60 min). Phage Z90 remained viable across psychrotrophic and mesophilic temperatures (<50 °C) and a wide pH range (3–12), suggesting high survival rates within the typical aquaculture conditions of temperature (10–25 °C) and pH (6.5–7.4) [44,45]. Additionally, genomic analysis confirmed the absence of genes encoding toxins, virulence factors, and antibiotic resistance in the phage Z90 genome, making it a safe candidate for practical application. Altogether, these biological characteristics and genomic safety profiles collectively endorse the phage Z90 as a promising biocontrol agent for aquaculture applications.

As the phage Z90 genome was composed of 234,057 bp dsDNA, it was classified as a jumbo phage. Its large genome may provide enhanced functional potential and broad applications. Compared to common phages, jumbo phages are reported to exhibit more autonomous life activities independent of the host bacterium, and their genomes encode RNAP for mRNA transcription and tRNA for protein translation [46]. Phylogenetic analyses and genome comparisons suggested that the phage Z90 belonged to the genus *Ferozepurvirus* within the family *Chimalliviridae*, whose members have been reported to assemble a proteinaceous phage nucleus for genome replication [47,48]. This nucleus-like structure shields the phage DNA from the host’s CRISPR–Cas system, reducing the likelihood of bacterial resistance and highlighting the promising application potential of *Chimalliviridae* phages [49,50,51]. However, the mechanisms by which jumbo phages protect their genome from DNA-targeting host defense systems at the early stages of infection (before nucleus formation) have not been fully explored. At this stage, the single-injected phage genome is highly vulnerable to host defenses, necessitating the rapid formation of a protective shell following infection [52]. Recent studies have reported the formation of membrane-associated intermediates, termed early phage infection (EPI) vesicles, during the initial stages of jumbo phage infection [47]. These vesicles are thought to act as transient protective compartments for the phage genome prior to the assembly of the proteinaceous phage nucleus, thereby shielding it from the host’s DNA-targeting defense systems. Mozumdar et al. [7,53] proposed that the EPI vesicles originate from the host inner membrane and are assembled from injected phage proteins, phage DNA, and host lipids. The EPI vesicles are also considered to be transcriptionally active, membrane-bound organelles, indicating their functional relevance during early infection [53]. In this study, the phage Z90 exhibited an approximately 3-log reduction in the infection titer after treatment with 5% chloroform. Therefore, we suspect that lipid-associated structures, possibly EPI vesicles, might be involved during Z90 infection, although further experimental evidence is required to confirm this hypothesis.

Phage Z90 exhibits strong lytic activity against the lethal *A. hydrophila* 2408 strain, demonstrating its potential as an effective antibacterial agent. However, its narrow host range limits its effectiveness in practical aquaculture systems, where multiple pathogenic bacterial species often coexist. Phage therapy using a single phage is often insufficient to control multiple pathogenic bacteria and can lead to bacterial resistance. To overcome these limitations, the development of phage cocktails and phage antibiotic combinations (PACs) has emerged as an important therapeutic strategy [54]. Phage cocktails can be used to cover strain-level heterogeneity, as well as to simultaneously treat multiple pathogens [55]. For example, Zhang et al. [56] formulated a three-phage cocktail that effectively lysed 64.93% of a diverse panel of 77 *Vibrio* strains, including *Vibrio parahaemolyticus*, *Vibrio alginolyticus*, *Vibrio vulnificus*, and *Vibrio anguillarum*, providing a promising biocontrol approach in seafood production. Kim et al. [57] designed PACs based on complementarity groups, which pair phages with non-overlapping receptor specificities. Using this strategy, they established three PACs that were effective against ≥96% of 153 clinical *Pseudomonas aeruginosa* isolates, including biofilm cultures, and showed comparable efficacy in an in vivo wound infection model, providing a blueprint for developing broad-spectrum PACs. Lu et al. [58] demonstrated that the combination of Shigella phages (SSE1, SGF2, and SGF3), lysozyme, and antibiotics (particularly β-lactam antibiotics) significantly increased the bactericidal activity against *Shigella* spp. In this study, the in vitro bactericidal assays showed that during co-culture of the phage Z90 (MOI = 100) with *A. hydrophila* 2408, the bacterial population initially declined sharply within the first 2 h and then rebounded slightly, eventually stabilizing at a low level (OD600 < 0.4). This dynamic likely reflects the onset of the phage lytic cycle and the emergence of phage-resistant subpopulations. However, an appropriate combination of the phage Z90 and ampicillin achieved complete inhibition of *A. hydrophila* within 12 h, demonstrating that Z90 can be effectively incorporated into PACs to enhance therapeutic efficacy and reduce antibiotic usage. These findings not only confirm that Z90 is a promising antibacterial agent against *A. hydrophila* 2408 but also highlight its role as a key component in combination therapies, providing an important framework for the development of effective phage cocktails and PACs in the future. In this context, future research is warranted to explore phage cocktails, potentially combined with antibiotics, to further enhance inhibition of diverse pathogens and improve therapeutic efficacy.

The phage administration method and dose are crucial for the efficacy of phage therapy in aquaculture, and the optimization of these parameters can reduce treatment costs and increase treatment efficiency [54]. In this study, both intraperitoneal injection and immersion of phage Z90 increased the survival rate of diseased American eels, demonstrating the feasibility of phage therapy in this species. Moreover, consistent with previous studies [32,59], the eel survival rate was higher in the injection group than in the immersion group. One possible explanation is that intraperitoneal injection delivers the phage Z90 particles more effectively to the target sites, thereby enhancing its therapeutic efficacy. The subsequent quantification of the phage particles showed that the phage Z90 concentration in the muscles of the phage injection group was significantly higher than that in the muscles of the phage immersion group on day 9. In addition, the superior therapeutic efficacy of the phage injection method over the phage immersion method may also be attributed to the longer survival period of the phage Z90 particles in the animal body than in the aquaculture water, as revealed by the phage quantification results of eel muscle tissues and aquaculture water. However, although intraperitoneal injection is effective, it is labor-intensive and impractical for large-scale application, particularly when handling a high number of specimens or very small animals. In this regard, immersion and oral administration represent more practical and efficient approaches for phage therapy in aquaculture. Kumari et al. (2023) [60] demonstrated that immersion delivery is feasible, although the effective phage concentration in water needs to be much higher than that required for injection, which is consistent with our observations. Besides, for pathogens colonizing the skin or external surfaces, immersion treatment may offer superior efficacy compared to injection. Oral administration is regarded as the most feasible approach owing to its low cost and minimal stress imposed on the fish. Nevertheless, limitations include reduced phage stability and the harsh acidic and proteolytic environment in the gastrointestinal tract, which may necessitate the use of coating strategies or the incorporation of acid neutralizers in the phage formulation [61]. Donati et al. (2021) [62] demonstrated that oral administration via phage-coated feed allows phages to reach fish organs, with stable gut concentrations indicating effective gastrointestinal transit. Recent pharmacokinetic evaluation in *Litopenaeus vannamei* challenged with *V. parahaemolyticus* showed that orally delivered phage Φvp140 reaches the intestine and hepatopancreas, which are the primary infection sites, and that repeated feeding maintains high phage titers in vivo. This evidence further supports the feasibility of oral phage delivery in aquaculture and indicates that continuous or high-dose administration may be necessary to achieve effective therapeutic outcomes under farming conditions [63]. Taken together, these findings indicate that oral administration should be considered an important research direction for phage Z90. Future work may focus on developing feed-based delivery strategies and determining effective dosing regimens, with the goal of translating Z90 into a practical therapeutic product and ultimately reducing antibiotic dependence in eel aquaculture.

## 4. Materials and Methods

### 4.1. Isolation and Identification of Bacteria Pathogens

Nine bacteria strains (Supplementary Table S1), including *A. hydrophila* 2408, were isolated from diseased American eels (*Anguilla rostrata*) from an aquaculture pond located in Xiamen, China, according to the method described by Bakiyev et al. [8]. Briefly, samples from rotten skin and abdominal hemorrhagic fluid of diseased American eels were collected, suspended in 30 mL sterile water, and then incubated for 1 h at 30 °C and 180 rpm. The suspension was serially diluted and evenly spread on Luria–Bertani (LB) agar plates. After 12 h of incubation at 30 °C, individual colonies were picked and streaked five times on LB agar plates to obtain pure bacterial strains. The isolated strains were taxonomically classified based on their 16S rRNA gene sequences and biochemical properties. Among the nine bacteria strains, *A. hydrophila* 2408 was selected for further investigation because of its known pathogenicity to eels. The whole genome of *A. hydrophila* 2408 was sequenced, and its virulence-associated genes were further identified using the Virulence Factors Database.

### 4.2. Phage Isolation, Purification, and Amplification

Using *A. hydrophila* 2408 as the host, phage Z90 was isolated from aquaculture water collected in Xiamen, China. The method for phage isolation and purification was adapted from established protocols [6]. Briefly, the early log-phase *A. hydrophila* culture (OD_600_ = 0.3) was added to an equal volume of 0.45 μm-filtered aquaculture water and incubated at 37 °C for 8 h with shaking to enrich *A. hydrophila* 2408nfecting phages [64]. The bacterial suspension was then subjected to 0.45-μm filtration and examined for the presence of phages by using the double-layer agar plating method. Briefly, the filtrate was serially diluted, mixed with log-phase *A. hydrophila*, and incubated for 10 min in the dark. The mixture was then added to 4 mL of LB soft agar (0.5%, ~50 °C) and poured evenly onto LB agar (1.5%) plates to form a bilayer. Plaque formation was observed after 12 h of incubation at 37 °C. Individual plaques were picked and purified five times by the double-layer agar plating method and stored in SM solution (0.01% (*w*/*v*) gelatin, 8 mM MgSO4, 50 mM Tris-HCl, and 100 mM NaCl, pH 7.5) at 4 °C in the dark.

The phages were amplified by the stepwise expansion of the phage–bacteria culture systems [44,65]. Briefly, 1 mL of phage stock was added to 30 mL of early log-phase host cells and incubated at 37 °C and 180 rpm until lysis. The lysate was centrifuged at 8000× *g* for 15 min at 4 °C, and the supernatant was filtered through a 0.45 μm filter to obtain the phage suspension. This step was repeated by gradually increasing the volume of the culture to 1 L. The resulting lysate was centrifuged at 8000× *g* for 10 min at 4 °C, and the supernatant was filtered through a 0.45 μm filter to obtain the phage suspension. The phage suspension was mixed with DNase I and RNase A (1 μg/mL final concentration) and incubated at 4 °C for 1 h. Thereafter, the phage suspension was mixed with NaCl (1 mol/L final concentration) and incubated at 4 °C for 1 h. The resulting mixture was then mixed with 10% (*w*/*v*) polyethylene glycol 8000 and incubated at 4 °C for 1–3 d. After centrifugation at 12,000× *g* at 4 °C for 60 min, the precipitate was resuspended in SM buffer. The phage suspension was then purified using the cesium chloride density gradient ultracentrifugation at 200,000× *g* and 4 °C for 24 h. The concentrated phage bands were extracted using a sterile syringe and dialyzed 3–5 times with sterile SM buffer. The resulting phage concentrate was stored at 4 °C in the dark.

### 4.3. Transmission Electron Microscopy (TEM)

Phage morphology was observed using TEM with phosphotungstic acid-negative staining [66]. Briefly, 10 µL of phage concentrate was placed on a 200-mesh copper grid and allowed to adsorb for 10–30 min in the dark. The samples were then stained with 1% phosphotungstic acid for 20 min and air-dried for 30 min. Lastly, the phage particles were examined by TEM (Hitachi HT-7800, Tokyo, Japan) at 80 kV. Polyhedral head and tail lengths were measured using ImageJ software version 1.53e (n = 5).

### 4.4. One-Step Growth Curve Assay

The *A. hydrophila* 2408 sample was incubated to early log-phase (OD_600_ = 0.3) at 37 °C and 180 rpm for 10 min. Thereafter, the *A. hydrophila* and phage Z90 suspensions were mixed at a multiplicity of infection (MOI) of 0.01 and incubated for 10 min in the dark to allow phage adsorption. The mixture was centrifuged at 8000× *g* and 4 °C for 5 min, and the pellet was resuspended in 1 mL of LB liquid medium. The centrifugation process was repeated twice to remove unabsorbed phage particles. The final 1 mL suspension was added to 5 mL of LB liquid medium and cultured at 37 °C and 180 rpm. Phage titers were determined at 20 min intervals over a 3 h period. This analysis was performed with three biological replicates (n = 3).

### 4.5. Determination of Optimal MOI

The optimal MOI of the phage Z90 was determined as previously described [59]. Briefly, early log-phase *A. hydrophila* culture was mixed with phage suspensions at varying MOIs (10^−3^, 10^−2^, 10^−1^, 1, and 10). The mixture was incubated for 10 min in the dark and centrifuged at 8000× *g* to remove unabsorbed phage particles. After incubation at 37 °C for up to 4 h, the mixture was centrifuged at 8000× *g* for 5 min and filtered through a 0.45-μm syringe filter. The phage titer was examined by using the double-layer agar plating method. The sample generating the highest titer of plaque-forming units (PFU) was considered to have the optimal MOI. This experiment was performed using three biological replicates (n = 3).

### 4.6. Determination of Temperature, pH, and Chloroform Stability

The temperature, pH, and chloroform stability of the phage Z90 particles were tested according to the method described by Cao et al. [59] with slight modifications. For the temperature stability assay, 1 mL of the phage suspension (in SM buffer, pH 7.5) was incubated at 10, 20, 30, 40, 50, 60, 70, and 80 °C for 1 h. For the pH stability assay, 100 μL of the phage suspension was mixed with 0.9 mL of phosphate-buffered saline (PBS) and incubated at pH 2.0, 3.0, 4.0, 5.0, 6.0, 7.0, 8.0, 9.0, 10.0, 11.0, and 12.0 at 37 °C for 1 h. For the chloroform stability assay, 1 mL of the phage suspension (in SM buffer, pH 7.5) was incubated with 50 μL of chloroform or water at 37 °C for 1 h. After incubation, the residual phage titer was determined. All experiments were performed using three biological replicates (n = 3).

### 4.7. Determination of Host Range

A total of 15 bacterial strains (listed in Table 1) were used to determine the host range of the phage Z90. Among them, 3 strains of *A. hydrophila* and 3 strains of *Aeromonas veronii* were collected from the Marine Culture Collection of China, and the other 9 strains were isolated from diseased eels in this study. The host range was determined using spot tests. Briefly, the 15 bacteria strains were cultured separately to early log-phase, mixed with LB soft agar (0.5%), and spread evenly on LB agar (1.5%) plates. Subsequently, 5 μL of the phage suspension (10^10^ PFU/mL) was spotted on the surface of the upper agar layer. Plaque formation was observed after 12 h of incubation at 37 °C. The experiment was performed with three biological replicates (n = 3).

### 4.8. Genomic Analysis of the Phage Z90

The genomic DNA of the phage Z90 was extracted by the phenol–chloroform method [67]. Briefly, the phage concentrate was mixed with proteinase K (100 mg/mL), Sodium dodecyl sulfate (SDS, 10%, *w*/*v*), and Ethylenediaminetetraacetic acid (EDTA, 0.5 mol/L, pH 8.0), and incubated at 65 °C for 3 h. Thereafter, the sample was thoroughly mixed with an equal volume of phenol/chloroform/isoamyl alcohol (25:24:1, *v*/*v*/*v*) and centrifuged at 12,000× *g* for 10 min. The supernatant was transferred to a fresh tube; this step was repeated twice. For purification of the phage DNA, the supernatant was mixed with chloroform/isoamyl alcohol (24:1, *v*/*v*) and centrifuged at 12,000× *g* and 4 °C for 10 min. The resulting aqueous phase was mixed with isopropanol and incubated at −20 °C for 12 h. The sample was centrifuged at 12,000× *g* and 4 °C for 5 min, and the precipitate was washed twice with 70% cold ethanol. The pellet was then air-dried, resuspended in 100 μL of Tris-EDTA buffer (10 mM Tris-HCl and 1 mM EDTA, pH 8.0), and stored at −80 °C. The concentration and purity of the extracted DNA were determined using a NanoDrop spectrophotometer (Thermo Fisher Scientific,, Waltham, MA, USA).

Sequencing of the phage genome was performed by Ruixing Bio-Tech (Shanghai, China). The DNA sequencing libraries were prepared using the NEBNext^®^ Ultra™ DNA Library Preparation Kit (NEB, Ipswich, MA, USA) and sequenced on the Illumina HiSeq 4000 platform. The raw data was processed using Trimmomatic v0.32 to obtain clean data and assembled using Velvet v1.2.03. The open reading frames (ORFs) were predicted using Glimmer3 v3.02 [68] and GeneMarkS v4.28 [69]. The predicted protein sequences were compared with the NCBI NR database by blastp v2.2.30+ for function annotation. Transfer RNAs (tRNAs) were identified using tRNAscan-SE v.2.0 (http://lowelab.ucsc.edu/tRNAscan-SE/, accessed on 15 January 2025).

BLASTn was used to identify Z90-related phages with the highest genomic similarity, and the relevant genome sequences were downloaded from GenBank. The genome sequences of the *Aeromonas*-infecting jumbo phages were also included for phylogenetic analysis. The phylogeny of Z90 and its related phages was analyzed using VICTOR [70]. The ViPTree server was used to generate a proteomic tree based on genome-wide sequence similarities [71], computed by tBLASTx. In addition, the genomic similarity between the phages was assessed using VIRIDIC at default parameters [72]. The genome of the *Aeromonas* phage Z90 reported here is available in the GenBank database under the accession number PV663807.1.

### 4.9. In Vitro Evaluation of Phage Bactericidal Effects

The phage suspension was mixed with 100 µL of early log-phase *A. hydrophila* cells (10^7^ CFU/mL) at different MOIs (0.01, 1, and 100) in a 96-well plate. Additionally, 100 µL of *A. hydrophila* cells was mixed with 100 µL of LB liquid medium (negative control, NC) or 100 µL of ampicillin (25 mg/mL; positive control, PC). The 96-well plates were incubated in the Varioskan LUX Multimode Microplate Reader (Thermo Fisher Scientific, Waltham, MA, USA) at 37 °C and 180 rpm for 12 h. The OD_600_ was measured every 0.5 h. The experiment was performed with three biological replicates (n = 3). 

The checkerboard screening method [73] was used to test whether phages have a potent combined bactericidal effect with ampicillin. Briefly, 50 µL of the phage suspension of different MOIs (0.01–100) was mixed with 50 µL of ampicillin at varying concentrations. The mixtures were then added into wells in a checkerboard manner. The wells were then inoculated with 100 µL of *A. hydrophila* (10^7^ CFU/mL), and incubated in a microplate reader at 37 °C and 180 rpm for 12 h. The OD_600_ was measured every 0.5 h during the incubation period. The bactericidal effects of antibiotics alone, phage alone, and untreated bacterial cultures (controls) were evaluated simultaneously. Each phage–antibiotic combination was tested in five wells (technical replicates), and the entire experiment was independently repeated with three biological replicates (n = 3).

### 4.10. In Vivo Evaluation of Phage Therapeutic Effects

To establish an acute *A. hydrophila* 2408 infection model, 20 American eels (18 ± 2 cm in length) were randomly categorized into four groups (n = 5/group)and intraperitoneally injected with different concentrations of *A. hydrophila* 2408 (3× 10^5^, 3× 10^6^, and 3× 10^7^ CFU/fish). The control group was injected with PBS. The eels were observed for infection symptoms and were immediately removed when they died. The mortality rate was recorded until 7 d post-infection (dpi).

To evaluate the in vivo efficacy of phage Z90 in controlling *A. hydrophila* infections, 50 American eels (18 ± 2 cm in length) were randomly divided into five groups (n = 10/group), namely the blank control group, NC group, antibiotic group, phage injection group, and phage immersion group. Eels in the blank control group were infected with *A. hydrophila*, while the eels in the other four groups were intraperitoneally injected with 0.1 mL of *A. hydrophila* 2408 (6 × 10^6^ CFU/mL). After 2 h of infection, eels in the NC group were injected with 0.1 mL of PBS, while those in the antibiotic and phage injection groups were injected with 0.1 mL of ampicillin (2.5 mg/mL) and 0.1 mL phage suspension (6 × 10^8^ PFU/mL), respectively. The eels in the phage immersion group were placed in aquaculture water containing the phage suspension (10^8^ PFU/mL final concentration). The clinical signs and mortality rates of the eels were recorded daily for 7 d. Phage concentrations in the aquaculture water were recorded daily by using the double-layer agar plating method. For each time point, three independent water samples (n = 3) were collected, with phage concentration determined for each sample using the double-layer plate method. Muscle tissues were aseptically collected from both the eels that died during the experiment and those that survived until the endpoint. Phage titers were analyzed by using the double-layer agar plating method. At the endpoint, muscle tissues were aseptically collected from three eels per group in the phage immersion and phage injection groups. Tissue phage titers were determined in triplicate for each fish (n = 3) using the double-layer agar method.

### 4.11. Statistical Analysis

Statistical analyses were conducted using GraphPad Prism (version 10). Data are expressed as the mean ± standard deviation (SD). Phage titers at each time point were compared between the water and chloroform treatment groups using one-way ANOVA.

Differences in muscle phage concentrations between groups of eels were evaluated using the Mann–Whitney U test. For in vitro bactericidal assays (Figure 6B,C,E), differences among groups were assessed using one-way ANOVA followed by Tukey’s posthoc multiplecomparison test. For in vivo survival experiments (Figure 7B), Kaplan–Meier survival curves were plotted and differences among groups were analyzed using the log-rank test. Significance thresholds were set at *p* < 0.05 and indicated in figures as ns = not significant; * *p* < 0.05; ** *p* < 0.01; *** *p* < 0.001; **** *p* < 0.0001.

## 5. Conclusions

In this study, we successfully isolated and characterized a novel lytic jumbo phage, Z90, targeting *A. hydrophila*. Its short latent period, large burst size, excellent lytic capacity, and safe genomic content highlight its potential as a promising candidate for phage therapy applications. Furthermore, the synergistic antibacterial activity observed when Z90 was combined with ampicillin further underscored its applicability in PAC strategies. In vivo therapeutic effects demonstrated that both intraperitoneal injection and immersion administration significantly improved the survival of *A. hydrophila*-infecting American eels, confirming the therapeutic feasibility and efficacy of phage therapy in this economically important aquaculture species. The comparison of different delivery routes provides practical guidance for future phage therapy strategies of Z90 in eel farming. Overall, as the first report of phage therapy for American eels, this study supports the potential of jumbo phages as promising bioagents in aquatic pathogen controls.

## Figures and Tables

**Table 1:** Virulence-associated genes identified in *A. hydrophila* 2408 genome.

VF Category	Related Genes
Nutritional/Metabolic factor	*allB*, *allS*, *argP*, *asbC*, *basB*, *basC*, *basD*, *basF*, *basG*, *bauA*, *bauB*, *bauC*, *bauD*, *bauE*, *bauE*, *bioA*, *bioB*, *bioD*, *bplA*, *carA*, *carB*, *ccmA*, *ccmB*, *ccmC*, *ccmE*, *ccmF*, *chuA*, *chuT*, *chuU*, *chuV*, *chuW*, *dhbE*, *dhbF*, *entA*, *entB*, *entC*, *entE*, *fbpC*, *feoB*, *fepA*, *fepC*, *fepD*, *fpvA*, *fpvI*, *hgpB*, *hitC*, *hpt*, *iroC*, *lbtC*, *lplA1*, *mgtB*, *mgtC*, *PA2383*, *panC*, *phzG1*, *ptxR*, *purCD*, *purM*, *pvcA*, *pvcB*, *pvdD*, *pvdE*, *pvdH*, *pvdI*, *pvdL*, *pyrB*, *shuA*, *shuU*, *shuV*, *shuY*, *ybtP*, *ybtQ*
Motility	*AHML_RS07540*, *cheA*, *cheA-2*, *cheB*, *cheB-2*, *cheD*, *cheR*, *cheR-3*, *cheV*, *cheV3*, *cheW*, *cheY*, *cheZ*, *eptC*, *flaA*, *flaG*, *flaH*, *flaJ*, *fleN*, *fleQ*, *fleR*, *fleR/flrC*, *fleS*, *fleS/flrB*, *flgA*, *flgB*, *flgC*, *flgD*, *flgE*, *flgF*, *flgG*, *flgH*, *flgI*, *flgJ*, *flgK*, *flgL*, *flgM*, *flgN*, *flgO*, *flgP*, *flhA*, *flhB*, *flhF*, *flh*, *fliA*, *fliC*, *fliE*, *fliF*, *fliG*, *fliH*, *fliI*, *fliJ*, *fliK*, *fliL*, *fliM*, *fliN*, *fliO*, *fliP*, *fliQ*, *fliR*, *flmD*, *flmH*, *flrA*, *flrB*, *flrC*, *lafK*, *motA*, *motC*, *motX*, *motY*, *nueA*, *nueB*, *PA1459*, *PA1464*, *PA3349*, *pdxJ*, *pomA2*, *pomB2*, *pseB*
Adherence	*cadF*, *cgsD*, *fimA*, *fimB*, *fimC*, *fimD*, *fimE*, *fimF*, *fimZ*, *frpC*, *gbpA*, *htpB*, *IlpA*, *lap*, *mam7*, *mshA*, *mshB*, *mshC*, *mshD*, *mshE*, *mshF*, *mshG*, *mshH*, *mshI*, *mshJ*, *mshK*, *mshL*, *mshM*, *mshN*, *mshO*, *mshP*, *mshQ*, *pebA*, *pilG*, *pilJ*, *pilR*, *psaC*, *rpoN*, *tapB*, *tapC*, *tapD/pilD*, *tapF*, *tapM*, *tapN*, *tapO*, *tapP*, *tapQ*, *tapT*, *tapU*, *tapV*, *tapW*, *tcpI*, *tppF*, *tsaP*, *tufA*, *vfr*, *yagX/ecpC*
Immune modulation	*acpXL*, *bexA*, *bplF*, *cap8J*, *cap8P*, *cpsA/uppS*, *cpsB/cdsA*, *cysC*, *ddrA*, *fabZ*, *flmK*, *FTT_RS04140*, *galE*, *galU*, *gmhA*, *gmhA/lpcA*, *gtrB*, *hisH2*, *htrB*, *kdkA*, *kdsA*, *kdtB*, *KP1_RS17330*, *kpsF*, *lgtF*, *lpxA*, *lpxA/glmU*, *lpxB*, *lpxC*, *lpxD*, *lpxH*, *lpxK*, *lpxL*, *lsgD*, *msbA*, *ompA*, *ompP2*, *opsX/rfaC*, *orfM*, *pbpG*, *pgi*, *pks1*, *pks15*, *ppsB*, *ppsC*, *rfaD*, *rfaE*, *rfbB*, *rfbC*, *rfbD*, *rffG*, *rpe*, *Rv2962c*, *ugd*, *waaA*, *waaF*, *waaQ*, *wbpK*, *wbpL*, *wbpM*, *wbtL*, *wbuZ*, *wcbN*, *wcbP*, *wcbT*, *wecA*
Effector delivery system	*AHA_RS09305*, *ascN*, *atsA*, *atsB*, *atsD*, *atsG*, *atsH*, *atsI*, *atsJ*, *atsK(Aerolysin A)*, *atsL*, *atsP*, *atsQ*, *atsS*, *bprB*, *BPS_RS26875*, *BPS_RS26880*, *bsaN*, *CBU_0270*, *CBU_1079*, *CBU_1434*, *CBU_1566*, *CBU_1594*, *clpB*, *dotA*, *dotU*, *exeA*, *exeB*, *exeC*, *exeD*, *exeE*, *exeF*, *exeG*, *exeH*, *exeI*, *exeJ*, *exeK*, *exeL*, *exeM*, *exeN*, *exsA*, *glgX*, *hcp1*, *ipaH4.5*, *lasA*, *lidL*, *lirB*, *LPG_RS00105*, *LPG_RS00200*, *LPG_RS14840*, *lpnE*, *PA1663*, *ricA*, *ssrA*, *ssrB*, *tagT*, *vasH*, *vasK/icmF*, *vgrG1*, *vgrG2*, *vgrG3*, *vipA*, *vipB*
Regulation	*bvgA*, *bvgS*, *bvrR*, *cdpA*, *csrA*, *devR/dosR*, *fur*, *letA*, *letS*, *mprA*, *phoP*, *phoQ*, *phoR*, *rcsB*, *relA*, *rpoS*, *sigA/rpoV*
Biofilm	*adeF*, *adeG*, *algB*, *algC*, *algQ*, *algR*, *algU*, *algW*, *algZ*, *bopD*, *uxS*, *mucD*, *mucP*, *pmlR/bspR1*, *vpsG*
Exotoxin	*acpC*, *aerA/act*, *clbF*, *clbG*, *clbK*, *clbS*, *cyaB*, *cylA*, *cylB*, *hlyA*, *hlyB*, *hlyD*, *nheB*, *plcD*, *rtxA*, *rtxB*, *rtxC*, *rtxD*, *tlh*, *toxA*, *zot*
Stress survival	*ahpC*, *clpC*, *clpE*, *clpP*, *katA*, *LPG_RS11000*, *recN*, *sodB*
Antimicrobial activity	*acrA*, *acrB*, *farA*, *farB*, *mtrC*, *mtrD*
Exoenzyme	*BAS_RS06430*, *hap/vvp*, *nagI*, *stcE*, *tlyC*
Invasion	*ibeB*, *ompA*
Post-translational modification	*Mip*, *prsA2*
Others	*aatC*, *acfB*, *icl*

**Table 2:** Host range of phage Z90.

Bacterial Species	Strain	Source	Lytic Ability
*Aeromonas hydrophila*	2408	diseased American eels (*Anguilla rostrata*) ^b^	+
*Aermonas hydrophila*	1A00007	Mullet (*Mugil cephalus*) ^a^	−
*Aermonas hydrophila*	1A00032	Starry flounder (*Platichthys stellatus)* ^a^	−
*Aermonas hydrophila*	1A00179	Yellowfin Seabream (*Acanthopagrus latus*) ^a^	−
*Aermonas veronii*	1A00013	Mud ^a^	−
*Aermonas veronii*	1A02245	Yellowfin Seabream (*Acanthopagrus latus*) ^a^	−
*Aermonas salmonicida*	1K03261	Yellowfin Seabream (*Acanthopagrus latus*) ^a^	−
*Aeromonas caviae*	FJML01	diseased American eels (*Anguilla rostrata*) ^b^	−
*Aeromonas encheleia*	FJML02	diseased American eels (*Anguilla rostrata*) ^b^	−
*Aeromonas allosaccharophila*	FJML03	diseased American eels (*Anguilla rostrata*) ^b^	−
*Acinetobacter piscicola*	FJML04	diseased American eels (*Anguilla rostrata*) ^b^	−
*Citrobacter arsenatis*	FJML05	diseased American eels (*Anguilla rostrata*) ^b^	−
*Exiguobacterium enclense*	FJML06	diseased American eels (*Anguilla rostrata*) ^b^	−
*Raoultella ornithinolytica*	FJML07	diseased American eels (*Anguilla rostrata*) ^b^	−
*Pseudomonas pharyngis*	FJML08	diseased American eels (*Anguilla rostrata*) ^b^	−

^a^ Strains isolated from diseased American eels (*Anguilla rostrata*) from aquaculture pond of Xiamen, China during 2024. ^b^ Strains collected from the MCCC (Marine Culture Collection of China).

## Data Availability

The complete genome sequence of Aeromonas phage Z90 was submitted to GenBank under the accession number PV663807.1. The whole-genome sequence of *Aeromonas hydrophila* 2408 is publicly available under the BioProject accession number PRJNA1264476, BioSample accession number SAMN48554309, and GenBank accession number JBNYWU000000000.

## Supplementary Materials

The following supporting information can be downloaded at: https://www.mdpi.com/article/10.3390/antibiotics15010027/s1, Table S1: Dominant bacterial strains isolated from diseased American eels (*Anguilla rostrata*), Supplementary Figure S1: Colony morphology of *A. hydrophila* 2408 cultivated on RS Medium, Supplementary Figure S2: Phylogenetic tree of *A. hydrophila* 2408 and other representative *Aeromonas* strains based on 16S rRNA sequences, Supplementary Figure S3: Whole-genome phylogenetic tree of *Aeromonas hydrophila* 2408 and related *Aeromonas* strains generated using the Type (Strain) Genome Server (TYGS, https://tygs.dsmz.de, accessed on 20 July 2025).
