# Supplementary material for: Isolation, Characterization, and Evaluation of a Lytic Jumbo Phage Z90 Against Aeromonas hydrophila in American Eels (Anguilla rostrata)
Source: Antibiotics (Basel). 2025 Dec 31;15(1):27. doi: 10.3390/antibiotics15010027 (PMC12837969; doi:10.3390/antibiotics15010027)
Supplement: Supplementary file 1 [file antibiotics-15-00027-s001.zip › Supplementary material 2.pdf]

Table S1 Dominant bacterial strains isolated from diseased American eels (*Anguilla rostrata*)

Bacterial species	Strain	Closest strain (16S rRNA gene)	Similarity (%)
Aeromonas hydrophila	2408	*Aeromonas hydrophila subsp.hydrophila* ATCC 7966	99.24
Aeromonas caviae	FJML01	*Aeromonas caviae* CECT 838	98.76
Aeromonas encheleia	FJML02	*Aeromonas encheleia* LMG 16331	99.11
Aeromonas allosaccharophila	FJML03	*Aeromonas allosaccharophila* CECT 4199	98.95
Acinetobacter piscicola	FJML04	*Acinetobacter piscicola* LW15	98.98
Citrobacter arsenatis	FJML05	*Citrobacter arsenatis* LY-1	99.08
Exiguobacterium enclense	FJML06	*Exiguobacterium enclense* NIO-1109	99.29
Raoultella ornithinolytica	FJML07	*Raoultella ornithinolytica* JCM 6096	99.44
Pseudomonas pharyngis	FJML08	*Pseudomonas pharyngis* BML-PP036	99.2

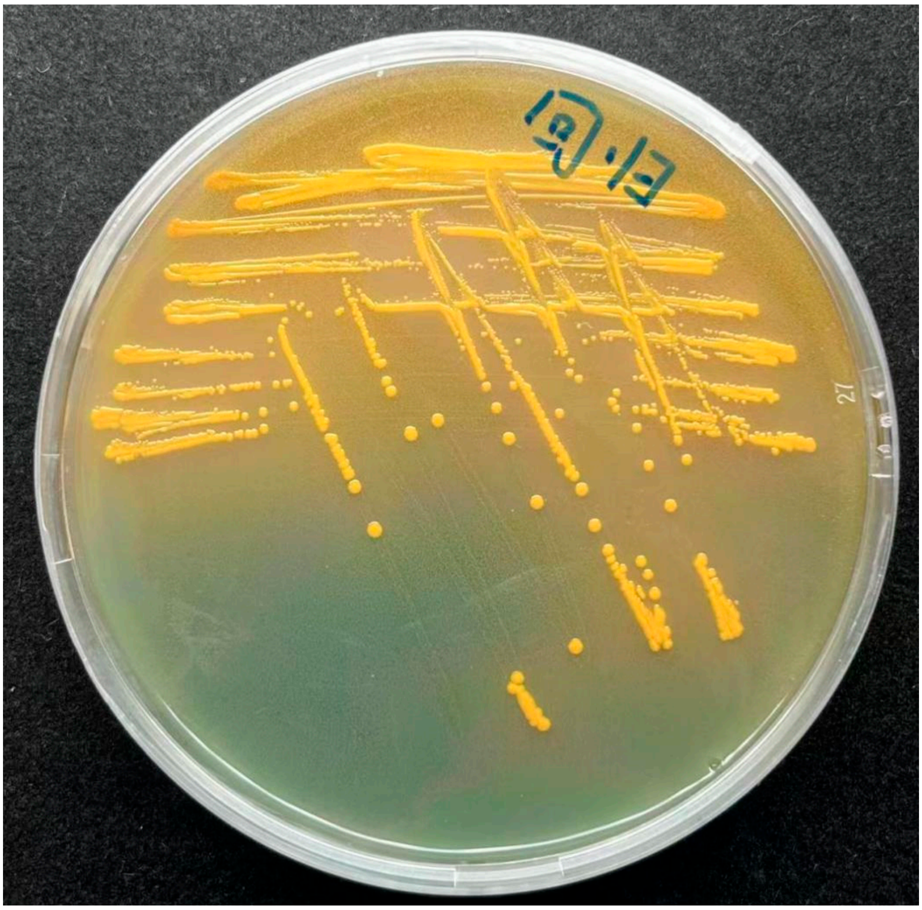

Supplementary Figure S1 Colony morphology of *A. hydrophila* 2408 cultivated on RS Medium

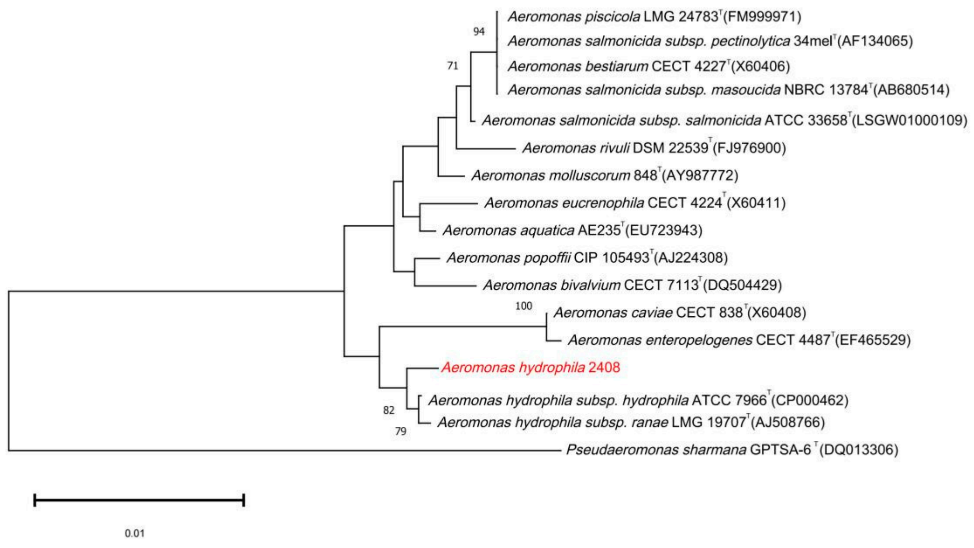

Supplementary Figure S2 Phylogenetic tree of *A. hydrophila* 2408 and other representative *Aeromonas* strains based on 16S rRNA sequences.

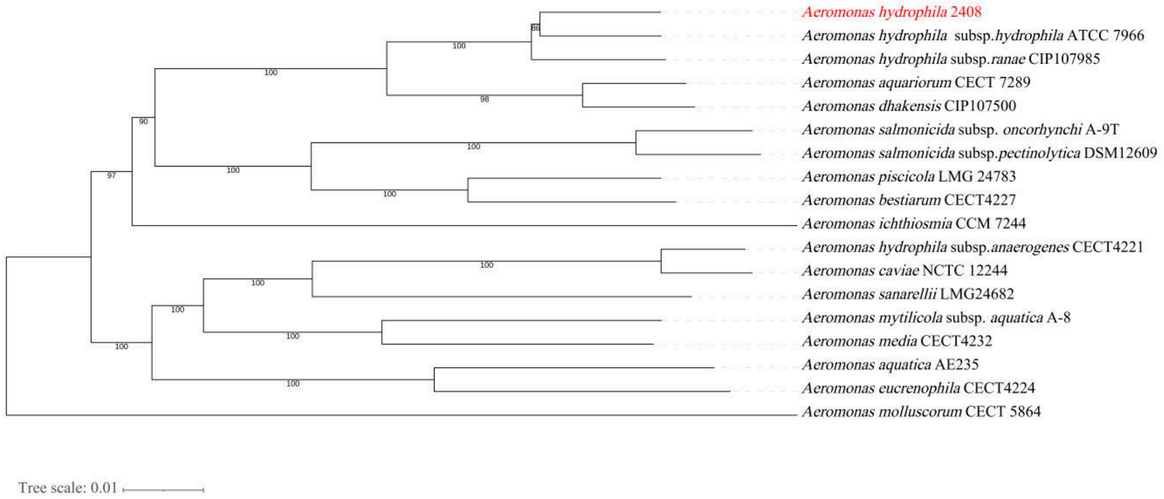

Supplementary Figure S3 Whole-genome phylogenetic tree of *Aeromonas hydrophila* 2408 and related *Aeromonas* strains generated using the Type (Strain) Genome Server (TYGS, https://tygs.dsmz.de)
